# Outcomes of STN-DBS in PD Patients With Different Rates of Disease Progression Over One Year of Follow-Up

**DOI:** 10.3389/fneur.2020.00600

**Published:** 2020-07-24

**Authors:** Renli Qi, Xin Geng, Baihui Huang, Ye Chen, Honggao Jiang, Yanghong Zou, Wei Wang, Yubin Li, Yushan Li, Lei Yin, Anxiong Liu, Xuelan Yang, Jinghui Li, Hualin Yu

**Affiliations:** Second Department of Neurosurgery, First Affiliated Hospital of Kunming Medical University, Kunming, China

**Keywords:** Parkinson's disease, deep brain stimulation, subthalamic nucleus, disease progression, outcome

## Abstract

Parkinson's disease (PD) is a progressive neurodegenerative disorder, and the rate of progression is different across individuals. Subthalamic nucleus deep brain stimulation (STN-DBS) has been shown to produce long-term symptom improvement in PD. In this retrospective study, we wanted to explore the effects of bilateral STN-DBS in PD patients with different rates of disease progression. Forty patients with PD were included. An index of progression rate was calculated by the ratio of the Unified Parkinson Disease Rating Scale, part III (UPDRS-III), score in the off-medication condition at baseline and disease duration. The patients were divided into fast-, medium-, and slow-progression groups by this index. The outcome measurements at the 1st, 6th, and 12th months after surgery were the changes in UPDRS-III scores in the off-medication/on-stimulation condition compared with the baseline. We found the following. (1). Motor functions in the different PD progression groups were improved by bilateral STN-DBS treatment at 1 year of follow-up. (2). However, compared to the slow- and medium-progression groups, the fast-progression group had less improvement at the 6th- and 12th-month follow-up. The results indicated that bilateral STN-DBS can improve motor functions of Parkinson's patients over the 1-year follow-up. Moreover, the outcomes in the slow- and medium-progression patients were better than those with fast-progression rates.

## Introduction

Parkinson's disease (PD) is a common neurodegenerative disorder characterized by the loss of dopaminergic neurons in the substantia nigra accompanied by clinical symptoms of bradykinesia, tremor at rest, rigidity, postural instability, asymmetric onset, and levodopa responsiveness (1, 2). Levodopa and dopamine agonists are the primary treatment for PD patients, but motor and non-motor complications and drug-induced dyskinesia will often appear 5–10 years after pharmacologic treatment in advanced PD patients (3).

PD is a progressive neurodegenerative disorder, and studies indicate that motor deterioration might progress linearly in proportion to disease duration (4–6). However, in the clinic, the slope of the progression is different across individuals, which may be related to the differential pathological involvement of CNS structures (7).

Subthalamic nucleus deep brain stimulation (STN-DBS) has been shown to produce long-term symptom improvement on motor and non-motor symptom in PD. According to some reports, the motor improvement induced by STN-DBS is sustained for up to 5–8 years after surgery, but some of the initial improvements, mainly regarding axial signs, progressively deteriorated (8, 9). However, the reported improvements of motor function vary from 40 to 70% in off-medication/on-stimulation conditions at the 12th months after surgery (9–11). There are limited reports about the factors that correlated with the efficacy of STN-DBS, but recent work has reported that it seems inappropriate to combine substantially different populations of patients—newly diagnosed, early fluctuators, or advanced dyskinetic individuals—within the same group to evaluate the efficacy of STN-DBS (12). Therefore, it is necessary to explore the effects of STN-DBS on PD patients with different rates of progression. In this study, we divided PD patients who had received bilateral STN-DBS treatment in our center into slow-, medium-, and fast-progression groups by an index of the rates of progression. The index was calculated by the ratio of UPDRS-III scores evaluated in the off-medication condition and disease duration before operation. We wanted to explore the effects of STN-DBS on PD patients with different progression rates by comparing the outcomes at the 1st, 6th, and 12th months after surgery.

## Methods

### Patient Enrollment

Forty patients from a single DBS center in the First Affiliated Hospital of Kunming Medical University who underwent bilateral STN-DBS surgery and whose locations of electrodes were verified by CT/MRI from 2015 to 2017 were enrolled in this retrospective cohort study. The diagnosis of PD followed the standard diagnostic criteria of the International Parkinson and Movement Disorder Society in 2015 (13). The inclusion criteria included (1) good levodopa response on Unified Parkinson Disease Rating Scale part III (motor) (improvement >30%), (2) drug-related complications (e.g., dyskinesia, or “on-off phenomenon”) even under optimal anti-parkinsonism medication adjustment, (3) absence of structural lesions in brain MRI, and (4) absence of dementia (mini-mental status exam >24) and active psychiatric diseases (depression). All patients provided written informed consent for STN-DBS surgery and for the study's evaluation procedure. This study was approved by the First Affiliated Hospital of Kunming Medical University Human Ethics Review Committee (No. 2016L46).

### Surgical Procedures

**Figure 1:**
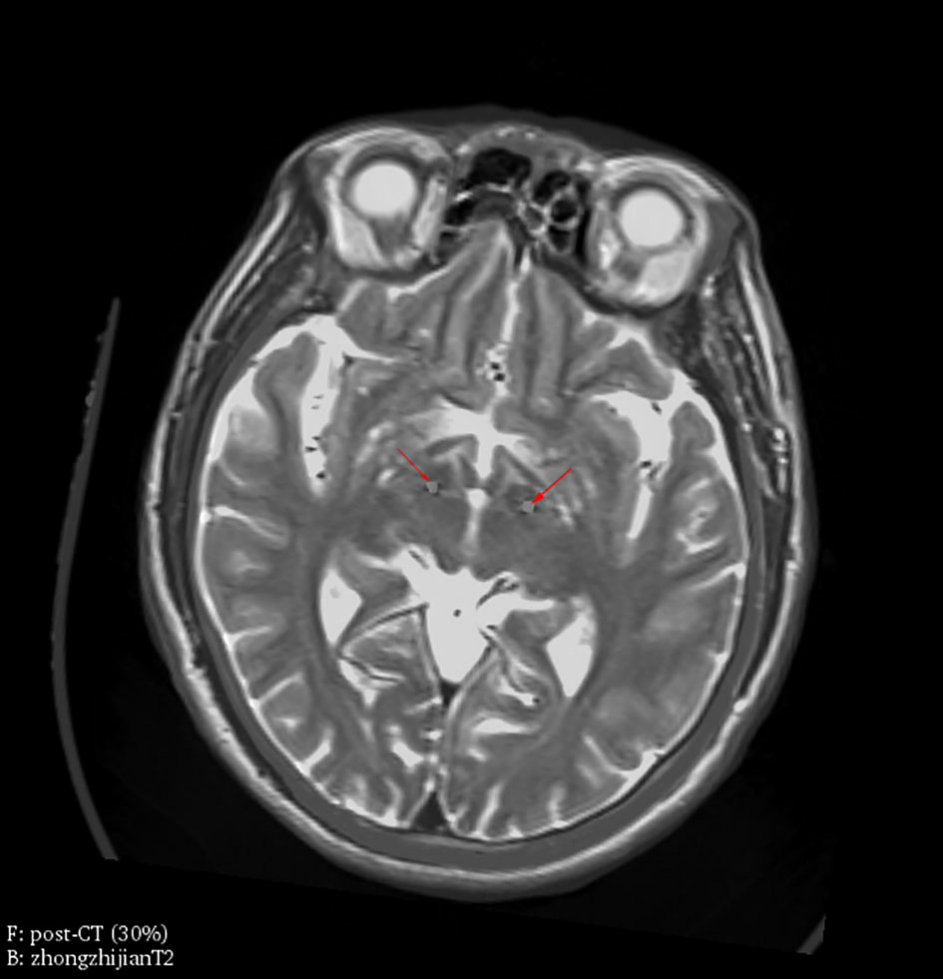
Electrode location was verified by fusion images of postoperative CT and preoperative MRI. Electrodes were located in bilateral STN (red arrowheads) in one representative patient, which is confirmed by fusion images of postoperative CT (30% transparency) as foreground and preoperative MRI (T2, 0% transparency) as background.

The surgical procedure comprised two phases. First, bilateral stereotactic STN implantation was performed under local anesthesia using MRI/CT image fusion for anatomical targeting. Images for targeting were obtained from a 1.5-Tesla magnetic resonance imaging (MRI) unit (Siemens, MAGNETOM Avanto, Germany). The standard settings for preoperative targeting included T1-weighted axial images (TR: 26 ms, TE: 6.9 ms, matrix size: 256 × 192, thickness: 0.7 mm) and T2-weighted axial images (TR: 4,800 ms, TE: 95 ms, matrix size: 256 × 192, thickness: 2.0 mm). Each of these sequences was performed in contiguous axial slices. A Leksell frame was used for the stereotactic procedure on the day of the operation. CT images were obtained with the patients' head in the frame. The images were transferred to a neuro-navigation workstation (SurgiPlan, Elekta, Sweden). Anterior commissure and posterior commissure (AC-PC) lengths were identified, and the tentative surgical target was set at the dorsolateral part of the STN. Quadripolar leads (Electrode model L301; PINS, Beijing, China) were implanted following the selected trajectory. Intraoperative electrophysiological recording (NeuroNav, Alpha Omega, Israel) and acute microstimulation were performed to evaluate clinical effects of implanted electrodes. Second, the pulse generator (Model G102R; PINS, Beijing, China) was then implanted in the right subclavicular area and connected through extension cables to the leads under general anesthesia. Postoperative CT was performed to confirm electrode positioning and to identify surgical complications. The electrode positions of enrolled patients in this study are all located in STN confirmed by fusion images of postoperative CT and preoperative MRI (Figure 1).

### Clinical Evaluation

Patients were evaluated at baseline and 1, 6, and 12 months after surgery. One month is the time when the stimulation generator was started to work after surgery. Baseline evaluations of motor symptoms (UPDRS-III) were performed in an off-medication condition after overnight withdrawal of anti-parkinsonian medication and the acute levodopa challenge test (ALCT) were performed in an on-medication condition after the administration of 1.5 times the usual L-dopa morning dose before the operation. After surgery, the scores on the UPDRS-III were evaluated in on-stimulation/off-medication conditions at approximately the 1st, 6th, and 12th months, at which time the patients returned for parameter modulation in the outpatient department in off-medication conditions. Evaluated symptoms included bradykinesia (items 23–26 and 31), tremor at rest (items 20 and 21), and rigidity (item 22) in UPDRS-III. The axial score evaluation included speaking, rising from a chair, gait, and postural instability (items 18 and 27–30) (10, 11). The L-dopa-equivalent daily dose (LEDD) was calculated according to recognized standard conversions (14).

### Statistical Analysis

The patients were divided into fast- (index ≥ 8), medium- (8 > index ≥ 5), and slow- (index <5) progression groups based on the index. For studies that have indicated that motor deterioration might progress linearly in proportion to disease duration (4–6), the index in this study was calculated by the ratios of UPDRS-III scores in the off-medication condition and disease duration before operation. Disease duration was from the onset of PD symptom to the time of UPDRS-III evaluation, and the onset of PD symptom is the time patients found the onset of mild tremor or leg drag, bradykinesia, and so on. The improvement in the acute levodopa challenge test was calculated by the ratio of the difference value between UPDRS-III scores in the off-medication condition and the most comfortable medication condition after the administration of 1.5 times the usual L-dopa morning dose and the scores in the off-medication condition. The improvement in motor function after surgery was evaluated by the ratios of the difference values between baseline score (off-medication) and scores in the on-stimulation/off-medication condition and baseline scores in off-medication.

All data were processed with the SPSS software package (version 21; SPSS, Chicago, IL). Group comparisons of clinical characteristics, including age, gender, disease duration, LEDD, improvement after the acute levodopa challenge test, indexes of progression rate, and UPDRS-III scores, were analyzed using one-way ANOVA for continuous variables. Repeated-measures ANOVA was used to examine the outcomes of STN-DBS on UPDRS-III scores or subscores at the 1st, 6th, and 12th months after surgery in each of the three groups. A *post hoc* comparison with Bonferroni correction was adopted when we compared the differences during groups or times. All *p*-values were two-tailed, and *p* < 0.05 was considered significant.

## Results

Forty patients were divided into slow- (index ≥ 8, *N* = 15), medium- (8 > index ≥ 5, *N* = 14), and fast- (index <5, *N* = 11) progression groups by the index of progression (IOP) rates. There were no differences in age, gender, LEDD, and improvement in the ALCT between the three groups. However, the fast-progression group had a shorter disease duration than the other two groups (both *p* < 0.001, one-way ANOVA). The UPDRS-III score of the fast-progression group evaluated in the off-medication condition was higher than that of the slow-progression group (*p* < 0.001, one-way ANOVA) but was not different from that of the medium-progression group (Table 1).

**Table 1:** Baseline demographics and clinical data in the slow-, medium-, and fast-progression groups.

	**SP group** **(*N* = 15)**	**MP group** **(*N* = 14)**	**FP group** **(*N* = 11)**	***t*** **or χ2** **(*p*-value), df**
Age, mean (SD)	60.93 (2.31)	63.93 (1.82)	65.73 (8.60)	1.16 (0.32), 39
Male, *N* (%)	10 (66.7%)	7 (50%)	7 (63%)	9.37 (0.01), 2
Duration, mean (SD)	10.8 (0.68)	9.36 (0.75)	5.09 (0.59)^*^^#^	16.71 (<0.001), 39
LEDD, mean (SD)	618.77 (72.72)	629.04 (75.49)	679.59 (83.43)	0.164 (0.85), 39
IOP, mean (SD)	3.39 (0.21)	6.48 (0.23)^*^^∧^	13.7 (2.07)^*^^#^	26.06 (<0.001), 39
ALCT improvement, mean (SD)	78.25% (3.09)	73.78% (2.68)	71.34% (4.55)	1.4 (0.26), 39
UPDRS-III scores, mean (SE)	36.47 (3.31)	59.79 (4.0)^*^	61.91 (5.09)^*^	12.82 (<0.001), 39

There were significant differences in disease duration, UPDRS-III scores, and IOP-values between slow-, medium-, and fast-progression groups but not for the factors age, gender, LEDD, and improvement after the acute levodopa challenge test. Note: UPDRS-III, the Unified Parkinson Disease Rating Scale, part III; LEDD, the levodopa-equivalent daily dose; IOP, the index of progression rate. SP, MP, and FP groups, slow-, medium-, and fast-progression groups.

“*”, p < 0.05 vs. the SP group;

“#”, p < 0.05 vs. the MP group;

“∧”, *p < 0.05 vs. the FP group*.

### The Motor Abilities Evaluated by UPDRS-III Before the Operation

The patients were evaluated by UPDRS-III in the defined off-medication condition before the operation. The total score and subscores of tremor at rest (items 20 and 21), rigidity (item 22), bradykinesia (items 23–26 and 31), and axial signs (items 18 and 27–30) were compared in three different progression groups. Both the total scores and the subscores (tremor, rigidity, bradykinesia, and axial) of the slow-progression group were significantly lower than those of the medium and fast groups (*p* = 0.001, 0.001, 0.016, 0.001, and < 0.001, respectively), however, there were no significant differences between the medium and fast groups (one-way ANOVA with Bonferroni *post hoc* test) (Table 2).

**Table 2:** The differences in motor ability in the slow-, medium-, and fast-progression groups.

**UPDRS-III scores(Med-off)**	**SP group** **(*N* = 15)**	**MP group** **(*N* = 14)**	**FP group** **(*N* = 11)**	***t* (*p*-value), df**
Total scores, mean (SD)	36.47 (12.8)	59.79 (14.81)^*^	61.91 (16.89)^*^	12.82 (<0.001), 39
Tremor, mean (SD)	5.73 (1.118)	11.57 (1.32)^*^	11.91 (2.17)^**^	5.54 (0.001), 39
Rigidity, mean (SD)	9.47 (0.79)	12.71 (0.90)	14.91 (2.23)^**^	4.42 (<0.016), 39
Bradykinesia, mean (SD)	13.07 (1.72)	22.43 (2.19)^*^	27.91 (4.27)^**^	7.85 (0.001), 39
Axial, mean (SD)	5.67 (0.73)	10.43 (1.18)^*^	11.91 (0.93)^**^	8.85 (0.001), 39

The total scores and subscores of UPDRS-III evaluated in the off-medication condition at baseline in the slow, medium, and fast groups. The subscores of tremor at rest (items 20 and 21), rigidity (item 22), bradykinesia (items 23–26 and 31), and axial signs (items 18 and 27–30) were compared in three different progression groups. This indicates that both total scores and subscores in the medium and fast groups are higher than in the slow group. SP, MP, and FP groups, slow-, medium-, and fast-progression groups. One-way ANOVA with Bonferroni post hoc test.

“*”, p < 0.05;

“**”, *p < 0.01 vs. the SP group*.

These results indicated that the motor ability of the slow group was significantly better than that of the medium and fast groups, but there was no difference between the medium and fast groups.

### The Outcomes of Bilateral STN-DBS at the One-Year Follow-Up

The UPDRS-III scores evaluated at baseline and at the 1st, 6th, and 12th months are shown in Table 3. These results showed the UPDRS-III scores evaluated at the 1st, 6th, and 12th months after surgery were significant lower when compared to the baseline in all three groups (all *p* < 0.001, paired *t*-tests).

**Table 3:** The UPDRS-III scores evaluated at baseline in off-medication and the 1st, 6th, and 12th months in off-medication/on-stimulation condition in the slow-, medium-, and fast-progression groups.

	**UPDRS-III scores, mean (SE)**			
**Group**	**Baseline**	**Post-op 1M**	**Post-op 6M**	**Post-op 12M**
SP	36.47 (3.31)	7.33 (1.02)	10.47 (1.61)	11.20 (1.59)
MP	59.79 (4.0)	13.36 (1.73)	18.64 (2.46)	17.80 (2.42)
FP	61.91 (5.09)	12.81 (2.68)	28.91 (4.60)	33.55 (4.01)

**Figure 2:**
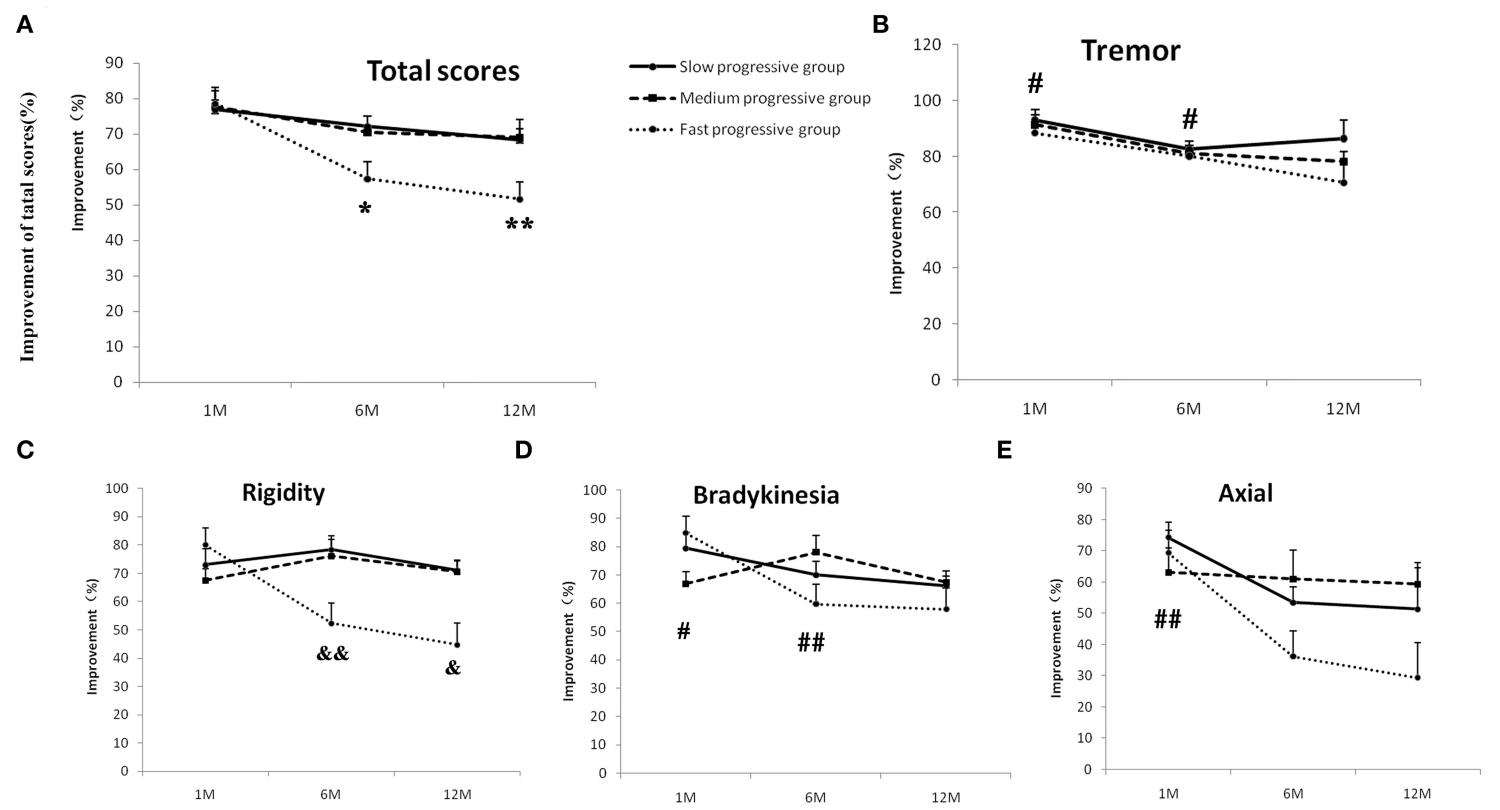
The outcomes of bilateral STN-DBS at stimulation onset (1 month) and 6 and 12 months in the slow-, medium-, and fast-progression groups. The effect of bilateral STN-DBS on the total motor scores **(A)**, resting tremor scores **(B)**, rigidity scores **(C)**, bradykinesia scores **(D)**, and axial scores **(E)** assessed by UPDRS-III in the three different groups. There are interaction effects between times and groups for total scores and rigidity scores. The *post hoc* test with Bonferroni correction indicated that the improvements of total motor function at the 6th and 12th months are significantly lower than that of the first month in the fast-progression group **(A)**, and the improvements of rigidity in the fast group are lower than in the slow-progression groups at 6th and 12th months **(C)**. There were no interaction effects between time and group on the resting tremor, bradykinesia, and axial scores. The *post hoc* test with Bonferroni correction indicated that the improvements of tremor and bradykinesia scores in the 12th month are lower than in the 1st and 6th months **(B,D)**. The improvement in axial tremor in the 12th month was lower than in the first month **(E)**. “*”/“**” indicates a significant difference from the first month in the fast-progression group, *p* < 0.05/0.01, respectively, “#” indicates a significant difference between the improvement of the 1st/6th and 12th months, *p* < 0.05/0.01. “&” indicates a significant difference between the improvement in the fast- and slow-progression groups, *p* < 0.05/0.01.

The outcomes of bilateral STN-DBS were measured by improvements in UPDRS-III motor scores in the off-medication/on-stimulation condition at the 1st, 6th, and 12th months after surgery, compared to the baseline (Supplemental Table 1). There are interaction effects between time (1st, 6th, and 12th months) and group (slow-, medium-, and fast-progression groups) on total UPDRS-III motor scores (*p* = 0.04, two-way repeated-measures ANOVA). For the group effect, there are no significant differences during three groups at three time points. For the time effect, there are significant differences in medium- (*p* = 0.017) and fast-progression groups (*p* < 0.001), but not in slow group. *Post hoc* tests with Bonferroni correction found that the improvements at the 6th and 12th months are significantly lower than that of the first month in the fast-progression group (*p* = 0.023 and 0.001) (Figure 2A).

The effects of bilateral STN-DBS on tremor, bradykinesia, and axial scores at the first, 6th, and 12th months in the slow-, medium-, and fast-progression groups were tested by two-way repeated ANOVA. There were main effects of time on the outcomes of tremor, bradykinesia, and axial scores (*p* = 0.004, 0.002, and 0.006, respectively), and there are no main effects of group or interactions between groups and times. A *post hoc* test with Bonferroni correction found that the improvement of tremor in the 12th month was lower than that in the first (*p* = 0.011) and 6th months (*p* = 0.03) (Figure 2B). The improvement of bradykinesia in the 12th month was lower than that in the first month (*p* = 0.02) and 6th month (*p* = 0.006) (Figure 2D). The axial outcomes in the 12th month were lower than in the first month (*p* = 0.008) (Figure 2E). There was an interaction between groups and times (*p* = 0.001, two-way repeated-measures ANOVA) on rigidity. For the group effect, there are significant differences at the 6th month (*p* = 0.001) and 12th month (*p* = 0.012); a *post hoc* test with Bonferroni correction found that the outcomes of STN-DBS on rigidity in the fast group are lower than the slow group both at the 6th (*p* = 0.008) and 12th months (*p* = 0.027) (Figure 2C).

These results indicated that the improvement in motor functions in the three groups was improved by bilateral STN-DBS treatment at the 1-year follow-up. However, the improvement in the fast progression group was not as good as in the slow and medium groups at the 6th and 12th months.

### The Factors Affect the Movement Improvement at 12th Month After Deep Brain Stimulation

The effects of clinical data, such as, age, gender, IOP, ALCT improvement, and LEDD, on the movement improvement at the 12th month were tested by multiple linear regression model using the stepwise method. We found that the regression model only included the IOP variable that had statistical significance (*F* = 12.575, *p* < 0.001, adjusted *R*^2^ = 0.229). The impact of IOP on the 12th-month improvement was significant (*p* = 0.001). The detailed results are shown in Table 4. The factors of age, gender, ALCT improvement, and LEDD have no significant effect on the improvement at the 12th month (all *p* > 0.5).

**Table 4:** The result of the multiple linear regression model.

**Variable**	**Coefficient**	**Standard error**	**Standardized** **coefficient**	**Significant**
Intercept	0.746	0.037		0.000
IOP	−0.015	0.004	−0.499	0.001

## Discussion

Previous work indicated that PD is a progressive disease, and the motor deterioration might progress linearly in proportion to disease duration (4–6); thus, the group information in this study was based on the index of progression rate, which was calculated by the ratios of the UPDRS-III scores in the off-medication condition and disease duration at baseline. Based on the group information, we found the following. (1). There were no differences in the LEDD and the improvement from the acute levodopa challenge test between the slow, medium, and fast groups at baseline. This finding may indicate that our patients in the fast group are not multiple-system atrophy with predominant parkinsonism (MSA-P), which would account for deterioration to greater severity and disability in a shorter time (15), accompanied by a poor response to levodopa (16). (2). The disease duration in the fast group was shorter than in the slow and medium groups, but the UPDRS-III score was higher than in the slow group, which means that the deterioration progressed very rapidly in the fast group. Previous studies have indicated that the progression rate in PD may be influenced by factors such as the onset age or complications associated with the disease. One study reported that an increase in the UPDRS-III score with similar disease duration was more pronounced in older patients than in younger patients (17). In another study, Burn and colleagues showed that the annual deterioration measured by the UPDRS-III score in PD patients with dementia was more severe than in PD patients without dementia (18). However, there were no significant differences in the onset ages among the three groups, and dementia was an exclusion criterion for our surgery.

In this study, we found marked improvement in motor function as evaluated by UPDRS-III scores in slow, medium, and fast groups at the 1st-, 6th-, and 12th-month follow-up. The improvements observed with bilateral STN-DBS in our study are in line with previous reports, which reported that the efficiency of treatment is approximately 40–70% in the off-medication/on-stimulation conditions at the 12th month after surgery (9–11). Furthermore, we found that the improvements in motor function in the fast group at 6 and 12 months were not as good as in the slow and medium groups at the same period. Maybe there are many reasons for the decline in improvement with passage of time. The multiple linear regression model found only that the regression model that included the IOP variable had statistical significance on the improvement of total UPDRS-III scores at 12 months, but adjusted *R*^2^ is low (0.229), which means the improvement at 12 months may have not linearly correlated with patients' IOP. We conduct Pearson correlation analysis between the improvement of total UPDRS-III scores at 12 months in three different groups and the clinical data before operation (IOP, gender, age, duration, UPDRS-III scores, ALCT improvement, and LEDD) (Supplemental Figure 1). The correlations are very poor between the improvement at 12 months and patients' gender (Supplemental Figure 1B), age (Supplemental Figure 1C), duration (Supplemental Figure 1D), UPDRS-III scores (Supplemental Figure 1E), ALCT improvement (Supplemental Figure 1F), and LEDD (Supplemental Figure 1G). The improvement lowly correlated with IOP of total groups (*R*^2^ = 0.25) but more highly correlated with IOP of the fast group (*R*^2^ = 0.49). Interestingly, the improvement has a good correlation to disease duration in the fast group (*R*^2^ = 0.61), which may indicate the faster progression and poorer improvement in the fast group.

It is interesting that there were no motor function differences according to the UPDRS-III scores between the fast and medium groups in the off-medication condition before the operation, but the outcomes following bilateral STN-DBS were quite different. Moreover, the motor functions showed significant differences between the slow and medium groups before the operation, but the outcomes following bilateral STN-DBS were similar. This result indicates that the pathogenesis of disease in the fast-progression group may be different from those of the slow and medium groups, but the detailed mechanism is not clear.

The lower improvements in motor function in the fast group may be the result of severe deterioration in PD patients in this group. One recent study suggested that disease severity plays a central role in the efficacy of STN-DBS in PD patients (12). Moreover, the progression of deterioration is quite different during individual PD patients (19). Our study implies that the fast progression of deterioration may counteract the partial outcomes of STN-DBS, so the improvement in motor function in the fast group is lower than in the slow- and medium-progression groups.

Many studies have reported efficiency outcomes of bilateral STN-DBS at different durations after surgery, from 6 months to 11 years (9, 20–23), and across different ages of disease onset, i.e., young-onset and old-onset PD patients (11). However, this is the first report of the outcomes of STN-DBS in PD patients with different progression rates. Some studies have also reported the outcomes of histo-pathologically proven MSA patients who underwent STN-DBS surgery because they were considered having PD at the time of surgery, and these studies found that clinical improvements were short-lasting (~6–12 months) and rapidly followed by the occurrence of disabling manifestations of MSA that counteracted the DBS benefits (24, 25). In our study, although the improvement in the fast-progression group was not as good as the slow and medium groups, the DBS benefit was significant when compared to baseline.

Some limitations of this study could be addressed in future research. First, the index of progression rate and the classification into slow, medium, and fast groups may not be very strict. Some researchers consider the progression of motor symptoms in medication-treated patients to be described in a linear model, but others think that the model is more complicated (5, 6, 19). Second, more comprehensive clinical data should be collected, such as UPDRS-I, II, and IV scores and outcomes in the on-medication/on-stimulation condition, to assess overall outcomes with STN-DBS.

In conclusion, our results supported the efficiency of STN-DBS for motor function in slow-, medium-, and fast-progression PD patients, but the outcomes for patients in the fast-progression group were not as good as those in the slow and medium groups. The different rates of outcomes could provide some guidance to neurosurgeons and neurologists when addressing the expectations of fast-progression patients before operations, as one study showed that addressing patients' expectations both preoperatively and postoperatively may play an important role in patient satisfaction and therefore in the overall success of STN-DBS surgery for Parkinson disease (26).

## Data Availability Statement

The datasets generated for this study are available on request to the corresponding author.

## Ethics Statement

The studies involving human participants were reviewed and approved by Medical Ethics Committee of Kunming Medical University. The patients/participants provided their written informed consent to participate in this study.

## Author Contributions

HY, JL, XG, and RQ designed and conducted the study, including patient recruitment, data collection, and data analysis. BH and YC prepared the manuscript draft with important intellectual input from HY and RQ. YZ, WW, HJ, YubL, YusL, LY, AL, and XY collected data and had complete access to the study data. YC and BH gave their editorial supports during the preparation of this manuscript. All authors approved the final manuscript.

## Conflict of Interest

The authors declare that the research was conducted in the absence of any commercial or financial relationships that could be construed as a potential conflict of interest.

## Supplementary Material

Supplemental Figure 1 The correlation between the improvements at 12th month and clinical data before operation. Pearson correlation analysis between the improvement of total UPDRS-III scores at 12 months and IOP **(A)**, gender **(B)**, age **(C)**, disease duration **(D)**, UPDRS-III scores **(E)**, ALCT improvement **(F)**, and LEDD **(G)**. Red dots represent the data of slow progression group; Green dots, medium group; purple dots, fast group. Black dashed lines are the correlation trend lines of total group, and the purple dashed lines are the trend lines of fast group.

Supplemental Table 1 The improvements with bilateral STN-DBS at stimulation onset (1 month), and at the 6th and 12th month in the three different groups.
