# Supplementary figures and images for: Outcomes of STN-DBS in PD Patients With Different Rates of Disease Progression Over One Year of Follow-Up
Source: Front Neurol. 2020 Jul 24;11:600. doi: 10.3389/fneur.2020.00600 (PMC7396496; doi:10.3389/fneur.2020.00600)

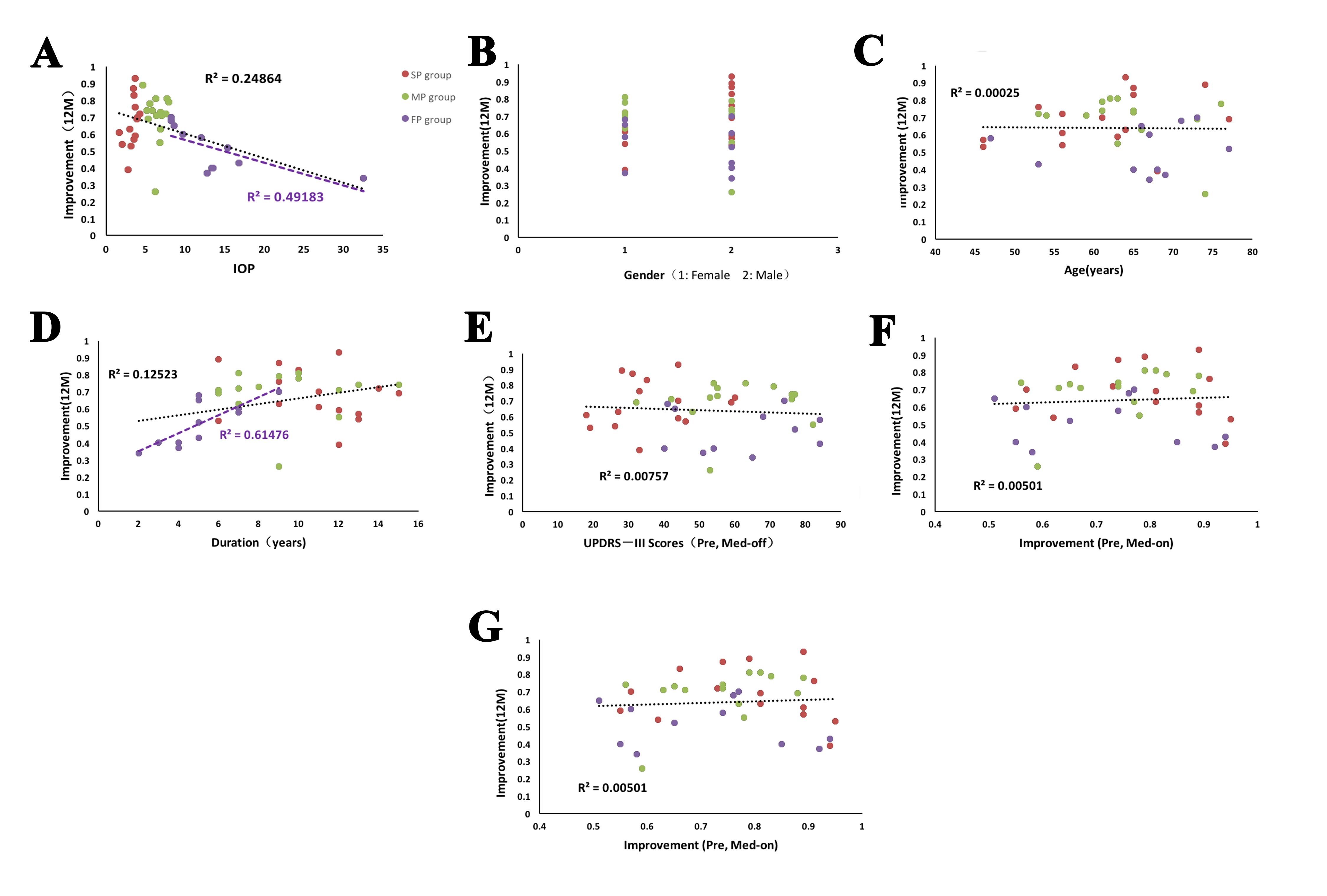

Supplement: Supplemental Figure 1 — The correlation between the improvements at 12th month and clinical data before operation. Pearson correlation analysis between the improvement of total UPDRS-III scores at 12 months and IOP (A), gender (B), age (C), disease duration (D), UPDRS-III scores (E), ALCT improvement (F), and LEDD (G). Red dots represent the data of slow progression group; Green dots, medium group; purple dots, fast group. Black dashed lines are the correlation trend lines of total group, and the purple dashed lines are the trend lines of fast group. [file Image_1.tif]
