# Supplementary material for: Outcomes of STN-DBS in PD Patients With Different Rates of Disease Progression Over One Year of Follow-Up
Source: Front Neurol. 2020 Jul 24;11:600. doi: 10.3389/fneur.2020.00600 (PMC7396496; doi:10.3389/fneur.2020.00600)
Supplement: Supplemental Table 1 — The improvements with bilateral STN-DBS at stimulation onset (1 month), and at the 6th and 12th month in the three different groups. [file Table_1.docx]

|  | **Group** | **Improvement**  **(1M), Mean (SE) %** | **Improvement**  **(6M), Mean (SE) %** | **Improvement**  **(12M), Mean (SE) %** |
| --- | --- | --- | --- | --- |
| **Total score** | **SP** | 76.82 (3.17) | 72.19 (2.87) | 68.36(3.52) |
|  | **MP** | 77.68 (2.75) | 70.39 (3.11) | 69.05 (3.66) |
|  | **FP** | 78.49 (4.74) | 57.38 (4.91) | 51.66 (4.04) |
| **Tremor** | **SP** | 93.08 (2.76) | 82.63 (6.42) | 86.47 (4.43) |
|  | **MP** | 91.41 (3.06) | 80.95 (3.56) | 78.18 (3.16) |
|  | **FP** | 88.3 (5.43) | 80.1 (6.50) | 70.71 (6.72) |
| **Rigidity** | **SP** | 72.99 (4.78) | 78.38 (3.48) | 71.09 (3.73) |
|  | **MP** | 67.52 (5.84) | 76.1 (3.96) | 70.5 (6.18) |
|  | **FP** | 79.98 (7.09) | 52.37 (7.48) | 44.83 (6.52) |
| **Bradykinesia** | **SP** | 79.42 (2.87) | 70.08 (5.46) | 66.17 (5.60) |
|  | **MP** | 66.98 (5.34) | 78.04 (5.49) | 67.43 (6.51) |
|  | **FP** | 84.79 (4.61) | 59.68 (7.76) | 57.91 (7.75) |
| **Axial** | **SP** | 61.62 (7.23) | 56.80 (11.79) | 50.86 (6.69) |
|  | **MP** | 63.59 (8.10) | 59.17 (8.91) | 53.68 (6.63) |
|  | **FP** | 67.62 (7.15) | 33.25 (10.64) | 22.94 (5.80) |
